# Draft Genome Sequence of Marine Actinobacterium Streptomyces spinoverrucosus SNB-032

**DOI:** 10.1128/MRA.00082-21

**Published:** 2021-04-22

**Authors:** Philipp Schwarzer, Stephan Flemming, John B. MacMillan, Andreas Bechthold

**Affiliations:** a Department of Pharmaceutical Biology, Institute of Pharmaceutical Sciences, University of Freiburg, Freiburg, Germany; b Department of Biochemistry, University of Texas Southwestern Medical Center, Dallas, Texas, USA; Georgia Institute of Technology

## Abstract

Actinobacteria represent a large source of diverse bioactive compounds of medical and economic importance. Here, we report the 8.8-Mb draft genome of the marine bacterium Streptomyces spinoverrucosus SNB-032. Bioinformatic sequence analysis proved similarities to known *Streptomyces* strains and revealed the capacity for the production of various secondary metabolites.

## ANNOUNCEMENT

The marine bacterium SNB-032 was isolated by Hu et al. (1) from a sediment sample from Trinity Bay, Galveston, Texas, in the course of an oncology screening program. A 16S rRNA sequence analysis revealed that SNB-032 had 99% identity to Streptomyces spinoverrucosus, which was collected from the atmosphere of Kuwait during a study of aerobic *Actinomycetales* strains and was described in 1982 (1, 2).

Fermentation of SNB-032 in a seawater-based acidic gauze medium [10 g starch, 4 g yeast extract, 2 g peptone, 1 g CaCO_3_, 40 mg Fe_2_(SO_4_)_3_·4H_2_O, 100 mg KBr] led to the production of the galvaquinones A, B, and C, 5,8-dihydroxy-6-isopentyl-2,2,4-trimethylanthra[9,1-*de*][1,3]oxazin-7(2*H*)-one, lupinacidin A, and islandicin (1).

The strain was provided on an agar plate and was cultured in the seawater-based acidic gauze medium as described above. From this, a laboratory cryostock was prepared for storage.

For genomic DNA (gDNA) isolation, the strain was cultivated in 20 ml tryptic soy broth (TSB) medium for 3 days. Subsequently, the cells were disrupted enzymatically (lysozyme, RNase A, and proteinase K), and proteins as well as RNA were precipitated. The gDNA was extracted by adding phenol-chloroform-isoamyl alcohol (25:24:1). Two volumes of isopropanol (100%) was laid on top of the aqueous phase, and gDNA was rolled up using a glass Pasteur pipette. The isolated DNA was washed in ethanol (70%), dried at 60°C for 10 min, and finally dissolved in 1 ml H_2_O.

The genome of SNB-032 was sequenced using the Pacific Biosciences (PacBio) single-molecule real-time (SMRT) technology following PacBio 20-kb template preparation using the BluePippin size selection system (Sage Science, Inc., USA) protocol; 7.5 μg of high-molecular-weight gDNA (final volume of 100 μl) was sheared using Covaris g-TUBES at 4,000 rpm for 60 s on each side. The sheared DNA was size selected with a BluePippin system using a cutoff range of 12 kb to 50 kb. The DNA damage repair, end repair, and SMRTbell ligation steps were performed as described in the template preparation protocol with the SMRTbell template preparation kit v1.0 reagents (PacBio). The sequencing primer was annealed at a final concentration of 0.2654 nM, and the P6v2 polymerase was bound at 0.1592 nM. The library was sequenced on a PacBio RS II instrument at a loading concentration (on plate) of 160 pM, using the MagBead OneCellPerWell loading protocol, DNA sequencing kit 4.0 v2, SMRT cells v3, and 4-h movies.

The resulting assembly contains 5 contigs with 8,854,993 bp. The largest contig is 7,827,506 bp, which represents 88% of the total assembly and the *N*_50_ value, because >50% of the bases are in this contig. The overall GC content is 70.9%, and the average coverage is about 40×. The quality was ensured with a spike-in control and it was confirmed that the well was not overloaded (P2 of <20%). HGAP (smrtanalysis_version = 2.3.0.140936.p5) was used for assembly.

Closely related strains were identified by sequence comparison of the 16S rRNAs via BLAST and included Streptomyces griseoviridis F1-27 (GenBank accession no. CP034687) (98.29% identity), *Streptomyces nigra* 452 (CP029043) (98.72% identity), and *Streptomyces cadmiisoli* ZFG47 (CP030073) (98.65% identity) (3).

For secondary metabolite analysis, the genome was analyzed using antiSMASH v5.0 (4). In total, 30 gene clusters were identified, including 10 that closely match (81 to 100%) known clusters of secondary metabolites. Two of these match known clusters of nonribosomal peptides (coelibactin [5] and scabichelin [6]), two match known clusters of terpenes (albaflavenone [7] and hopene), and one matches the cluster of a lanthipeptide (informatipeptin [8]). Furthermore, SNB-032 includes gene clusters that match polyketide synthase type II secondary metabolites; in addition to a gene cluster for spore pigments, the whole gene cluster for rishirilide A and B biosynthesis was identified. The biosynthesis of these secondary metabolites was intensively investigated by our working group (9–11). Three secondary metabolites were termed “others,” including a gene cluster for the biosynthesis of a γ-butyrolactone, desferrioxamine B, and desferrioxamine E, as well as ectoine, a natural compound that is produced by bacteria that live under extreme conditions (12).

### Data availability.

This whole-genome shotgun project has been deposited at DDBJ/ENA/GenBank under the accession no. JADPZX000000000. The version described in this paper is version JADPZX010000000. The Sequence Read Archive (SRA) data are available under the accession no. SRR13435345.
